# Effectiveness of Space Spraying on the Transmission of Dengue/Dengue Hemorrhagic Fever (DF/DHF) in an Urban Area of Southern Thailand

**DOI:** 10.1155/2012/652564

**Published:** 2012-02-08

**Authors:** Suwich Thammapalo, Supaporn Meksawi, Virasakdi Chongsuvivatwong

**Affiliations:** ^1^Office of Disease Prevention and Control 12, Department of Disease Control, Ministry of Public Health, Songkhla 90000, Thailand; ^2^Faculty of Health and Sports Science, Thaksin University, Phatthalung 93110, Thailand; ^3^Epidemiology Unit, Faculty of Medicine, Prince of Songkla University, Songkhla 90112, Thailand

## Abstract

Timely and extensive space spraying has been widely used to prevent the spread of dengue fever/dengue hemorrhagic fever (DF/DHF). Field evaluations on its effectiveness have been rarely reported. This study aimed to evaluate the timeliness, coverage, and effectiveness of space spraying for DF/DHF control using a geographic information system (GIS). Longitudinal monitoring of DF/DHF cases and spray activities in Songkhla municipality was done between May 2006 and April 2007. After a case was detected, subsequent cases occurring within a 100 meter radius of the index case's house and between 16–35 days of onset were considered as potential secondary cases. During the study period, 140 cases of DF/DHF were detected. Of these, 25 were identified as secondary infections from 20 index cases. Where a secondary infection occurred, the mean attack rate was 2.7 per 1,000 population. Two significant predictors for being a secondary case were both related to the house of the index case, namely, absence of window screens and being constructed with corrugated iron sheets. Our findings suggest that space spraying in the study area was inadequate and often failed to prevent secondary cases of DF/DHF. Control programs should target houses constructed with corrugated iron sheets.

## 1. Introduction

Dengue fever (DF), dengue hemorrhagic fever (DHF), or dengue shock syndrome (DSS) is one of the most important mosquito-borne viral diseases caused by one of four closely related, but antigenically distinct, virus serotypes (DENV-1, DENV-2, DENV-3, and DENV-4), of the genus *Flavivirus*. The virus is maintained in a cycle that involves humans and *Aedes aegypti*, transmitted by an infected female mosquito that is primarily a daytime feeder and mainly bites in the morning or late in the afternoon [1]. The transmission cycle starts when the female *Aedes *mosquito takes blood from a person during the viraemic phase (acute febrile) of illness and then becomes infected with the dengue virus. After an extrinsic incubation period of 8 to 12 days [2], the salivary glands of the mosquito become infected, and the virus is transmitted when the infective mosquito bites and injects the salivary fluid into the blood of another person. Following an incubation period in humans (intrinsic incubation period) of 3 to 14 days (average of 4 to 7 days) [2], there is often a sudden onset of the disease, with fever, headache, myalgias, loss of appetite, and a variety of nonspecific signs and symptoms, including nausea, vomiting, and rash. Infection with dengue viruses may produce a spectrum of clinical illnesses ranging from asymptomatic and nonspecific viral syndrome to severe and fatal hemorrhagic disease. Viraemia is usually present at the time of or just before the onset of symptoms and lasts an average of 5 to 9 days after the onset of illness. Symptoms caused by dengue infection may last 3 to 10 days, with an average of 5 days [3], after the onset of symptoms. This is the crucial period when the patient is most infective for the vector mosquito and contributes to maintaining the transmission cycle if the patient is not protected against vector mosquito bites. Infection with one of four dengue virus serotypes does not provide long-term cross-protective immunity, so a person living in a dengue-endemic area can have four dengue infections from their lifetime.

The trend of DF and DHF has increased epidemic dengue activity and increased incidence. DF and DHF are distributed in most tropical and subtropical area, in which 968,564 cases reported from 65 countries worldwide [4]. Associated risk factors for DHF outbreaks have focused on environmental factor, housing conditions, and human density. Environmental factors such as increased temperatures, numbers of rainy days, relative humidity, and rainfall [5, 6] have been found to be associated with DHF incidence. The transmission of dengue virus is sensitive with a seasonal variation since temperature changes affect vector-borne viral disease transmission and epidemic potential by the vector's reproductive rate, biting rate, and length of the extrinsic incubation period (EIP) [7], which increases from 3 days under 32°C to 14 days when the temperature is 20°C [8]. Other environmental factors, including housing conditions such as solid waste disposal problems, inadequate water supply, and absence of window screens [9], are also known to affect transmission rates. Virus transmission also increases with human population density. Urbanization in tropical countries has resulted in both a propagation of *Ae. aegypti* and an increase in the number of susceptible human hosts. In cities, the movement of viraemic persons is a more important means of transporting dengue viruses than movement of *Ae. aegypti* mosquitoes [10]. Vector control is the most effective method for killing vectors as quickly as possible and to reduce vector density. Control methods include modification or manipulation of environmental factors with a view to preventing or reducing vector proliferation and human-vector-pathogen contact.

Space spraying, spreading of microscopic droplets of insecticide in the air to kill adult mosquitoes, is an emergency control measure when an outbreak of dengue has occurred [11]. The standard operational guidelines recommended by WHO for an area with a surveillance system in place is to spray within a radius of 100 meters of affected houses and within 24 hours after receiving the case notification. Spraying should also be repeated at 7–10 day-intervals. A parous rate, the number of gravid female mosquitoes captured per house per person, of 10% or less within two days after spraying indicates that the spraying has been effective [12]. However, even with the implementation of widespread space spraying, the global DHF incidence has dramatically increased. Songkhla province in southern Thailand is a high endemic area for DF/DHF even though space spraying for dengue prevention and control was implemented since 2002. The annual incidence rate of DF/DHF in Songkhla province reported from the Bureau of epidemiology unit, Thailand during January–April in the year 2009 was 55 per 100,000 population compared to the national average of 11.7 per 100,000 population [12]. This may reflect the failure of outbreak control programs. Evaluation of the effectiveness of space spraying in terms of coverage and timeliness is very important, but very few studies have been done to date. This study aimed to evaluate the effectiveness of space spraying for DF/DHF in Songkhla municipality, southern Thailand during May 2006 to April 2007 and to determine the risk factors for secondary DF/DHF cases.

## 2. Materials and Methods

The secondary transmission was evaluated by estimated overall reproduction number (secondary case) of DF/DHF using a matrix model under the spatial-time condition of case.

### 2.1. Case Definitions

**Primary Case**

A new case that occurred in the community during the follow-up period.

**Index Case**

A primary case who infected another person living within a radius of 100 meters from the index case's house (an estimated daily flying distance of *Aedes* mosquitoes [13]).

**Secondary Case**

A case who resided within a radius of 100 meters from index case's house and developed symptoms between 16 and 35 days after diagnosis of the index case (calculated the minimum lag time for developing secondary case from summation of minimum of infectious period; *L*_min_ = 5 days, extrinsic incubation period; EIP_min_ = 8 days, and intrinsic incubation period; *I*_min_ = 3 days and the maximum lag time from a summation of maximum of infectious period; *L*_max_ = 9 days, extrinsic incubation period; EIP_max_ = 12 days, and intrinsic incubation period; *I*_max_ = 14 days [2, 3]).

**Coindex Case (Coprimary Case)**

A case that developed symptoms within 16 days after diagnosis of an index case and could be a possible source of infection for the secondary case.

**Secondary Attack Rate**

The number of secondary cases and susceptible persons residing within a radius of 100 meters of the index case's house during a specified time period.

### 2.2. Study Design

This is a longitudinal study monitoring cases of DF/DHF in an urban area of southern Thailand. The study was conducted from 1 May 2006 to 30 April 2007.

### 2.3. Study Setting

The municipality of Songkhla, which was selected to be the study site, is located at 07°12^'^N, 100°36′E on the east coast of southern Thailand, approximately 1,000 kilometers south of Bangkok. The municipality covers 9.3 km^2^ and lies on a small peninsular bordered by Songkhla Lake on the west side and the Gulf of Thailand on the east. There were 27,898 households with a 2006 midyear population of 92,032. The main occupations in this region consist of retail, fisheries, and government services. The population density on the north-eastern coast is low because this area contains beaches, government offices, schools and an airport. The central and western areas are densely populated by businesses involved in fisheries (western) and various types of retail stores (central).

In the past 5 years, the annual number of cases in Songkhla municipality has varied from 66 to 1,352 cases with an average annual incidence rate of 505 per 100,000 population. Within this municipality, there is one general hospital (Songkhla Hospital) containing 600 beds. This is the hospital where most serious DHF cases in the municipality are admitted since the next closest tertiary care hospital is 30 kilometers away.

### 2.4. Surveillance and Response Systems

All doctors taking care of patients in all hospitals in Thailand are regularly updated on management of DF/DHF cases. Definitions of suspected, clinically diagnosed, and serologically confirmed cases follow the World Health Organization (WHO) guidelines. Clinically diagnosed cases of DF, DHF, and DSS are compiled weekly at each hospital with a standard form containing essential information on patients. These reports are sent to provincial health officers who take local control measures and forward the information to the Department of Epidemiology in Bangkok. Consequently, local health officers implement control measures, such as space spraying, larva control, and health education to reduce disease transmission.

### 2.5. Data Collection

Data collection was consisted of 3 parts.

**(1) Hospital-Based Surveillance System for DF, DHF, and DSS Cases**

DF, DHF, and DSS cases were clinically diagnosed and serologically confirmed by physicians following the WHO guidelines. Each day, a field researcher verified all new cases admitted to both inpatient and outpatient wards of Songkhla Hospital from 1 May 2006 to 30 April 2007 and notified the response teams to carry out space spraying. The demographic data of each patient, location of their house, onset of illness, diagnosis date, and notification date were recorded.

**(2) Monitoring the Activities of Space Spraying**

Space spraying is usually performed for prevention of disease outbreaks or after notification of a case in order to interrupt disease transmission. In this study, we focus on spraying for DF/DHF after a case notification. Information on case notification date, date of spraying, address of index case, and neighboring houses which are sprayed were recorded by the rapid response teams.

**(3) Graphical Information System (GIS) Data**

A map of Songkhla municipality at the household level was obtained from The Songkhla Statistics Office. A house survey was carried out to verify existing houses and update new or nonexistent ones. The updated map was digitized and computerized at the GIS southern center.

### 2.6. Statistical Analysis

The distances, *D*_*ij*_, between houses belonging to pairs of cases in the study (one pair contains an index case and a nonindex case) were calculated using the following formula:

(1) $$D_{ij}=\sqrt{{\left(x_{i}-x_{j}\right)}^{2}+{\left(y_{i}-y_{j}\right)}^{2}},$$

where *x*_*i*_ and *y*_*i*_ represent the coordinates of an index case's house and *x*_*j*_ and *y*_*j*_ represent the coordinates of a nonindex case's house.

Twenty cluster of an index case were used in the calculation of relative secondary attack rate of 100 meter radius. Poisson regression was used to determine factors associated with the number of secondary DF/DHF cases with adjustment for age, sex, and whether or not an index case's house contained window screens. Variables with *P*-values less than 0.05 were considered significant. All analyses were performed using R software.

## 3. Results

### 3.1. General Characteristics of DF/DHF Cases

Occurrence of DF/DHF cases in Songkhla municipality was found throughout the year, with a seasonal variation demonstrated and a peak occurrence in January. The highest number of episodes (2,818 and 16 times) were reported in January 2007, December, and November 2006, respectively, while the least number of episodes (3 times) was found in April 2007.

**Figure 1:**
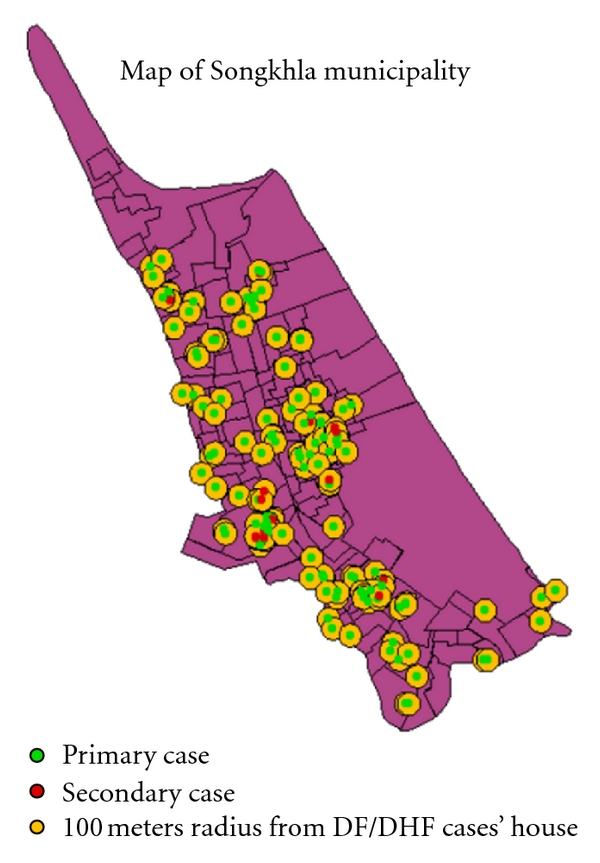
The distribution of DF/DHF case.

140 cases (69 DF cases and 71 DHF cases) were reported from Songkhla Hospital and the Provincial Health Office from 1 May 2006 to 30 April 2007. These DF/DHF cases were composed of 64 males (45.7%) and 76 females (54.3%). The median age was 10 years, and the most common age bracket was 10–15 years (38.8%). All cases were identified under the definition given above; 115 primary cases were classified into 20 index cases and 95 primary cases (which included 6 possible coindex cases). A further 25 secondary cases were also identified (Figure 1). Secondary cases were composed of 13 (52%) DF and 12 (48%) DHF cases, 14 (56%) cases were female, and the median age was 11 years. The primary cases were composed of 56 (48.7%) DF and 59 (51.3%) DHF cases, 62 (53.9%) cases were female, and the median age was 10 years (Table 1). There were no statistically significant differences in these general characteristics between primary and secondary cases.

45.0% of index cases with secondary cases (group 1) and 51.7% of index cases without secondary cases (group 2) were diagnosed as DHF. Most of the cases in both groups were female and the median age of group 2 was slightly higher than group 1 (Table 2).

### 3.2. Distribution of DF/DHF Secondary Cases

On average, secondary cases developed symptoms 28 days (±5 days) after the notification date of the index case. The mean distance between the index cases houses and the houses of the secondary cases was 40 meters (range: 10.2–94.3 meters).

### 3.3. Secondary Attack Rate

The relative secondary attack rate ranged from 1 to 6.3 per 1,000 population, with an average of 2.7. The highest attack rate was found in Toa-It community which has a high population density. Most of the workforce in this village consists of laborers, factory workers, or fishermen.

### 3.4. Environmental Characteristics of Index Case Houses

Table 3 shows a comparison of the environmental characteristics between the houses belonging to group 1 and group 2. Terraced houses were the most common type in both groups (group 1 = 70%, group 2 = 58.3%). Most houses of group 1 were constructed with mixed concrete (35.5%) or wood and concrete (35.0%) while most houses of group 2 were constructed with concrete only (56.7%). 15% of group 1 houses were constructed of corrugated iron sheets, but only 5% in group 2. 85.0% of group 1 houses and 52.5% of group 2 houses had no window screens, respectively.

Table 4 shows a comparison of house index and container index between the two groups of index cases. There were plenty of breeding sites for *Aedes* mosquitoes found from the larva survey, such as cement tanks in the toilet areas, water jars, and discarded containers. The mean house index among group 1 was 73.4 (sd = 17.4) and among group 2 was 66.9 (sd = ±29.1). The mean container index for group 1 was 30.8 (sd = 12.2) and among group 2 was 30.1 (sd = 19.5). No statistical differences between these two groups were found.

### 3.5. Coverage and Timeliness of DF/DHF Control Activities

Space spraying and larva control were implemented after a case was notified. Among houses in group 1 and group 2, due to time constraints, only 40% and 40.8% were surveyed for mosquito larva, respectively. Deltacide was used inside all of the houses from group 1. Among group 2 houses, incomplete spray was the result due to operational difficulties. 94.2% were sprayed inside, and 5.8% were sprayed only in front of the house. The coverage of spraying for houses in group 1 was 21.89 meters (1,788 m^2^) and 23.85 meters (1,506 m^2^) for houses in group 2, which is approximately 22 times less than the coverage recommended by WHO (100 meters or 31,428.4 m^2^). In terms of timeliness, spraying was initiated within a median of 17.3 hours after receiving case notification for both groups.

### 3.6. Factors Affecting Occurrence of Secondary DF/DHF Cases

Univariate analysis demonstrated that factors associated with secondary DF/DHF infection were age of index case, house construction material and use of window screens (Table 5).

Multivariate Poisson regression confirmed the association for male gender (IDR = 2.7) and house constructed with corrugated iron sheets (IDR = 4.0) after adjusting for age, and use of window screens (Table 6).

## 4. Discussion

Findings from this study showed ineffective space spraying measures during the study period. WHO recommendations were not properly followed, and this may have interfered in the final outcome. Twenty-five secondary infections were identified despite timely spraying. The relative secondary attack rate was as high as 6.3 per 1,000 population with an average of 2.7 indicating a failure in outbreak control. The average spraying area was small compared with WHO recommendations. Space spraying was implemented only once after case notification. Incomplete spraying in terms of both coverage and time may cause *Aedes* mosquitoes to emigrate from untreated areas into the previously sprayed areas. Koenraadt et al. [14] determined, that 7 days after insecticide spraying, 50% of the original number of mosquitoes were reestablished by extend around 50 meters or 15 meters within 2 days of spraying.

 The results of this study demonstrate that sex of the secondary case and index case houses constructed with corrugated iron sheets was significantly associated with secondary infections. Males had a 2.7 times higher risk of being infected compared to females. This observation merits further investigation with a larger sample and analysis of sex-specific behaviors that might modify risk of infection. Houses constructed with corrugated iron sheets played a significant role in transmission of DF/DHF in the community. This type of housing material cannot prevent mosquitoes from entering a house, where unsuspecting residents are easy prey for hungry mosquitoes. Temperatures inside houses constructed with corrugated iron are higher than houses constructed with concrete or wood. It is known that the extrinsic incubation period (EIP) is shorter at higher temperatures; a 5-day decrease in the EIP may triple the virus transmission rate [15]. The warmer temperatures inside a corrugated iron house may allow vectors to survive and reach maturity more rapidly thus promoting a quicker transmission of the virus compared to houses constructed with other materials. Additionally, window screen was a significant predictor for transmission in univariate analysis but turned to be nonsignificant in multivariate analysis. This confounding could be explained by the association of window screen and type of house or roof. Thus, the general construction of house may be more important than whether the house has window screen. The exact mechanism needs further investigation.

The first limitation in this study concerns the calculation of the number of secondary infections. We assumed that the minimum lag time or the minimum period in the developing secondary case from summation of minimum of infectious period; *L*_min_, extrinsic incubation period; EIP_min_, and intrinsic incubation period; *I*_min_ (*L*_min _ + EIP_min _ + *I*_min_) and the maximum lag time from a summation of *L*_max_, EIP_max_, and *I*_max _  (*L*_max _ + EIP_max _ + *I*_max_). So, the variation of number of secondary case may be due to the distinction in each period of this assumption. Secondly, the movement of viraemic persons was not determined. The movement of persons is a means of carrying or receiving the dengue virus to places that may encourage disease spreading; a case identified in the community could have been an indigenous one or an imported one.

## 5. Conclusion

Our findings suggest that space spraying in the study area was inadequate and often failed to prevent secondary DF/DHF infections. Our recommendations are to increase the spray area to cover a radius of 100 meters from the index case's house and to repeat spraying 7–10 days after the initial spray. Control programs should target houses constructed with corrugated iron sheets.

## Figures and Tables

**Table 1:** General characteristics of DF/DHF cases.

Characteristics	Type of case		*P* value
	Primary (%)	Secondary (%)	
Type of dengue			
DF	56 (48.7)	13 (52.0)	0.94
DHF	59 (51.3)	12 (48.0)	
Sex			
Male	53 (46.1)	11 (44.0)	0.97
Female	62 (53.9)	14 (56.0)	
Age group			
Median	10	11	0.6

**Table 2:** General characteristics of DF/DHF cases classified by group of index cases.

Characteristic	Index case group		*P* value
	With secondary cases (group 1) (%)	Without secondary cases (group 2) (%)	
Type of dengue			
DF	11 (55.0)	58 (48.3)	0.76
DHF	9 (45.0)	62 (51.7)	
Sex			
Male	13 (35.7)	51 (42.5)	0.24
Female	7 (64.3)	69 (57.5)	
Median age (yrs)	9	10.5	0.1

**Table 3:** Comparison of environmental characteristic of index case houses.

Factor	Index case group		*P* value
	With secondary cases (group 1) (%)	Without secondary cases (group 2) (%)	
House type			0.36
Terraced	14 (70.0)	70 (58.3)	
Townhouse	0 (0.0)	4 (3.3)	
Single house	3 (15.0)	22 (18.3)	
Slum house	3 (15.0)	20 (16.7)	
Apartment	0 (0.0)	4 (3.3)	
Construction material			0.05
Concrete only	7 (35.0)	68 (56.7)	
Concrete and wood	7 (35.0)	33 (27.5)	
Wood only	3 (15.0)	13 (10.8)	
Corrugated iron sheet	3 (15.0)	6 (5.0)	
Window screens present			0.005
No	17 (85.0)	63 (52.5)	
Yes	3 (15.0)	57 (47.5)	
Garbage piles around house			0.71
No	18 (90.0)	111 (92.5)	
Yes	2 (10.0)	9 (7.5)	
Piped water			
No	3 (15.0)	13 (10.8)	0.59
Yes (noncontinuous flow)	2 (10.0)	11 (9.2)	
Yes (continuous flow)	15 (75.0)	96 (80.0)	

**Table 4:** Comparison of house and container index.

Factor	Index case group		*P* value
	With secondary cases (group 1) (%)	Without secondary cases (group 2) (%)	
House index (hi)			0.36
[0,30]	0 (0.0)	10 (8.8)	
[30,100]	107 (100)	103 (91.2)	
Median	72.5	70	
Container index (ci)			0.89
[0,30]	9 (45.0)	56 (49.6)	
[30,100]	11 (55.0)	57 (50.4)	
Median	31.3	29.9	

**Table 5:** Univariate analysis of factors affecting incidence of secondary DF/DHF.

Variable	IDR	95% CI	*P* value
Male gender	2.21	0.88–5.54	0.08
Age of index case (years)	0.90	0.80–1.00	0.04
House type: ref. = single house			
Terraced	1.90	0.50–6.60	0.3
Slum house	2.30	0.50–11.4	0.3
House material: ref. = concrete			
Mixed concrete and wood	2.37	0.83–6.77	0.1
Wood	1.81	0.47–7.00	0.39
Corrugate iron sheet	5.04	1.30–19.5	0.02
Window screen (no versus yes)	4.10	1.20–14.1	0.01
Pipe water: ref. = continuous flowing			
None	1.40	0.40–4.70	0.62
Noncontinuous flowing	1.00	0.20–4.20	0.97
Garbage pile near the house	1.60	0.40–7.10	0.53
House index	1.01	0.99–1.03	0.11
Container index	1.00	0.99–1.03	0.42
Time lag of spraying (hours)	0.99	1.00-1.01	0.51
Area of spraying (m^2^)	1.00	1.00-1.00	0.45

**Table 6:** Multivariate analysis of factors affecting incidence of secondary DF/DHF.

Variables	Crude IDR (95% CI)	Adj. IDR (95% CI)	*P*-value
Age of index case (years)	0.9 (0.8–1)	0.9 (0.8–1)	0.10
Male gender	2.2 (0.9–5.5)	2.7 (1–7.2)	0.04
House construction: ref. = Concrete			
Mixed concrete and wood	2.4 (0.6–5)	1.7 (0.6–5.4)	0.30
Wood	1.8 (0.5–7)	0.8 (0.2–3.4)	0.74
Corrugated iron sheets	5.0 (1.3–19.5)	4.0 (1.0–16.8)	0.05
No window screens	0.4 (0.1–1.2)	0.5 (0.1–1.8)	0.30
Time lag of spraying (hours)	1.0 (0.8–1.2)	1.0 (0.8–1.3)	0.90
Area of spraying (m^2^)	1.0 (1.0-1.0)	1.0 (1.0-1.0)	0.68
